# *Saccharomyces cerevisiae* Diversity in *Arbutus unedo* L. Fermentations in Association with the Volatile and Sensory Similarities of the Distillates

**DOI:** 10.3390/foods11131916

**Published:** 2022-06-28

**Authors:** M. Margarida Baleiras-Couto, Ilda Caldeira, Filomena Gomes, Goreti Botelho, Filomena L. Duarte

**Affiliations:** 1 INIAV IP.—Instituto Nacional de Investigação Agrária e Veterinária, Pólo de Dois Portos, Quinta de Almoínha, 2565-191 Dois Portos, Portugal; ilda.caldeira@iniav.pt (I.C.); filomena.duarte@iniav.pt (F.L.D.); 2 BioISI—Biosystems and Integrative Sciences Institute, Faculty of Sciences, University of Lisbon, 1749-016 Lisbon, Portugal; 3 MED—Mediterranean Institute for Agriculture, Environment and Development, Institute for Advanced Studies and Research, Universidade de Évora, 7006-554 Évora, Portugal; 4 CERNAS—Research Centre for Natural Resources, Environment and Society, Polytechnic Institute of Coimbra, Coimbra Agriculture School, 3045-601 Coimbra, Portugal; fgomes@esac.pt (F.G.); goreti@esac.pt (G.B.)

**Keywords:** indigenous yeasts, strawberry tree fruit, alcoholic fermentation, distillation, volatile composition

## Abstract

The fermentation of *Arbutus unedo* L. fruit is traditionally carried out in the production of spirits. The present study followed the spontaneous fermentation of *A. unedo* fruit harvested in October and December 2019 by two producers from the central region of Portugal. The microbiota was studied, and although a great diversity of indigenous yeasts was found, *S. cerevisiae* isolates could still be grouped into eight clusters, and a good separation between producers was achieved. Based on the results of a multivariate analysis of the physical-chemical and volatile composition of the distillates, a distinction between the distillates from the two producers was determined. Moreover, these findings are corroborated by the similarities in flavor that were found. Along with the variability found in the distillates, *S. cerevisiae* isolates could be clustered and associated with each producer. On the other hand, the differentiation of the harvesting period was not so clear. The characterization of the indigenous yeasts associated with the fermentation process of *Arbutus unedo* L. fruit can serve as an important contribution to the preservation of the specific characteristics of its distillates.

## 1. Introduction

The strawberry tree (*Arbutus unedo* L.), which belongs to the Ericaceae family, is an autochthonous plant of the Mediterranean–Atlantic region. In Portugal, the *A. unedo* L. is a forest species present in most of the continental territory.

In recent years, *A. unedo* has been recognized as a species of interest for economic and ecological purposes. The interest in establishing orchards for fruit production has increased due to their higher yields in relation to the harvest of naturally regenerating plants in forested areas. In addition, these plants have also been studied as important sources of bioactive compounds [1]. Recent works have shown that establishing clones adapted to the relevant agro-ecological conditions, the use of fertilization, and the preservation of organic soil layers contribute to the sustainability of this agroforestry system, the quality of the fruit, and production [2]. As a consequence of recent investments in the establishment of new orchards, Portugal is currently the world’s largest producer of *A. unedo* fruit [3]. Similar work has been carried out in Turkey, where a program allowed for the selection of strawberry tree genotypes of high quality from the native strawberry tree population that grows in the central Black Sea Region of Turkey [4].

Ripe fruit is normally harvested in autumn in successive harvests over a period of 2–3 months. The fruit can be consumed fresh although this is not common, probably because the fruit only reaches a pleasant flavor if consumed when it is overripe [5]. Most commonly, strawberry tree fruit is used in the production of jams and jellies, vinegars, liqueurs, and spirits after undergoing a fermentation process [6]. In Portugal, the distilled beverage called “Aguardente de Medronho”, is the product with the most economic interest [6].

Previous work has analyzed and compared the distillates obtained from fermented fruit harvested from different seedlings and clonal plants [7]. The results obtained suggested that the main differences found among the distillates could result from the alcoholic fermentation process, with autochthonous microbiota being more important for the volatile profile of each sample than are those of clonal or seedling origin.

The process of the alcoholic fermentation of *Arbutus unedo* L. fruit is normally carried out spontaneously, exclusively by indigenous yeasts, with a sequence that includes non-*Saccharomyces* species, followed by the dominance of the *Saccharomyces cerevisiae* and *Pichia membranaefaciens* species [8]. Traditionally, fermentation occurs without temperature control and can last for several months. The harvested fruit is immediately transported and sorted (with the removal of unripe fruit, peduncles, and leaves) in order to avoid undesirable microbial growth, and it is then placed in plastic or stainless-steel tanks [9]. Inside the tanks, the fruit can be crushed, and drinking water is usually added, depending on the rainy season before the harvest. A maximum of 3 L of water per 15 kg may be added, depending upon the initial moisture in the fruit. This procedure increases the distribution of nutrients, leading to the better development of the yeasts present, and it decreases the probability of oxidation due to the lesser amount of contact between the fermented fruit masses and oxygen [9]. The tanks where the fermentation takes place have a lid to seal them and a valve, which both controls the release of the carbon dioxide produced during the fermentation and prevents air from entering, so as to avoid unwanted oxidation [9].

Despite all of the work carried out with *A. unedo* fruit and its distillates, studies on the autochthonous microbiota present during the alcoholic fermentation of this fruit are still lacking. In the present work, the industrial-scale processes of fermentation and distillation of two commercial producers from central Portugal were followed. Two deposits from each producer—one with fruit collected in October and another one with fruit collected in December—were selected for future sampling. The microbiota from the fermentation of *A. unedo* fruit were analyzed, and the yeast isolates were characterized to detect the *S. cerevisiae* isolates, which were further typed by SSR markers to evaluate species diversity. Furthermore, the distillations were processed separately, both under industrial conditions, followed by the determination of the volatile composition and the analysis of the sensory similarities of the distillates.

## 2. Materials and Methods

### 2.1. Experiment and Sampling

The orchards where the *A. unedo* fruit was hand-collected belong to two private producers located in central Portugal: Producer 1 (P1) in Estreito, a municipality of Oleiros (39.94118 N; −7.80801 W), and Producer 2 (P2) in Signo Samo, a municipality of Pampilhosa da Serra (40.027699 N; −7.922814 W). The biophysical characterization of both orchards is described in Table 1. Both orchards are established upon soils classified as dystric Leptosols and Cambisols [10] formed from the sedimentary and metamorphic shale rocks known as “Complexo xisto-gresoso das Beiras” [11].

Fruit harvests took place during between October and December in the autumn of 2019.

Plastic tanks of 120-liter capacity were filled with approximately 90 kg of fresh *A. unedo* fruit, without crushing the fruit or inoculating it with yeast, as is the traditional procedure used by commercial producers. The tanks were closed with air locks filled with water, and spontaneous alcoholic fermentation happened at room temperature (around 13–14 °C) in a dark place without vibration.

Two deposits from each producer—one with fruit collected in October, and the other with fruit collected in December—were selected for future samplings.

Samples of mashed, fermented fruit were collected in duplicate from each deposit during two periods, December 2019 (T1) and February 2020 (T2). Samples were collected in aseptic bags, hermetically sealed, immediately placed in a refrigerated container, and transported to the laboratory.

The temperature and Brix degree were measured for each deposit at the sampling times, T1 and T2, using, respectively, a glass thermometer and a digital refractometer.

### 2.2. Microbiological Analysis

The microbiological analysis of the fermented fruit, in reference to the total microbial counts, yeast counts, and acetic bacteria counts, were carried out in the laboratory just after their arrival. Samples were shaken by hand before serial dilutions were performed. The culture media used were as follow: plate count agar (PCA) for the growth of all viable microorganisms; yeast extract, peptone, and dextrose (YPD) for the growth of yeast (with chloramphenicol and biphenyl added to suppress bacterial and fungal growth, respectively); glucose, yeast extract, and carbonate (GYC) for the growth of acetic bacteria (with pimaricin and penicillin added to suppress the growth of yeast and lactic acid bacteria, respectively) [12].

The enumeration of microorganisms was performed in duplicate using the spread-plating technique after appropriate serial decimal dilution. Plates were incubated at 25 °C for at least 48 h for yeast and acetic acid bacteria and at 30 °C for total microorganisms.

### 2.3. Yeast Isolation

A total of 20 yeast colonies grown in yeast growth media (10 yeast colonies per replicate sample) were collected randomly. Each isolate was further purified in YPD media. The codes used to identify isolates are listed in Table 2. Each isolate is identified with a letter followed by a number (from 1 to 20).

### 2.4. Saccharomyces cerevisiae Characterization and Differentiation

#### 2.4.1. Cell Lysis

Cell lysis was performed by suspending a loop of colony mass in 30 μL of NaOH (20 mM) in a PCR tube. The tubes were placed in a thermocycler (PCR), applying a temperature of 99 °C for 10 min. The suspension was then placed on ice and used immediately or frozen for future use.

#### 2.4.2. Primer Specific for *S. cerevisiae*

The cell lysates of all of the isolates were analyzed using a *S. cerevisiae*-specific PCR amplification primer pair (Sc1), which generated a fragment of around 301 bp of product [13]. The amplified products were separated by agarose gel electrophoresis and compared with the amplified product from a reference strain of *S. cerevisiae* so as to exclude non-*Saccharomyces* isolates.

#### 2.4.3. Microsatellite Analysis with SSR Markers

Only the isolates which generated the *S. cerevisiae* amplicon were further characterized by means of 11 microsatellite loci: C3, C5, C4, C6, C8, C11, SCAAT1, ScAAT3, ScAAT5, YKL172w, and YPL009c [14]. Two multiplex reactions were performed.

Multiplex PCR for Mix 1 was performed using 5 SSR loci labelled with different WellRED fluorescent dyes. The PCR amplification conditions were as follows: 10 μL of the mixture consisting of Qiagen (X2) (5 μL), H_2_O (1.925 μL), DNA from a yeast isolate (1 μL), and an F + R primer solution with a final concentration of 0.1 μM for primers C8 and C5, 0.15 μM for C11 and Sc3, and 0.025 μM for C3. Multiplex PCR for Mix 2, using 6 SSR loci labelled with different WellRED fluorescent dyes, consisted of Qiagen mix (X2) (5 μL), H_2_O (1.05 μL), DNA (1μL), and an F + R primer solution of YKL172w, YPL009c, C6, Sc1, C4, and Sc5, with final concentrations of 0.1 μM, 0.15 μM, 0.05 μM, 0.1 μM, 0.25 μM, 0.1 μM, and 0.1 μM, respectively.

The amplification was carried out on a TGradient 96 thermocycler (Biometra) using different programs. For PCR Mix 1, the initial step was performed at 94 °C for 15 min, followed by 40 cycles each of 30 s at 94 °C, 90 s at 56 °C, and 60 s at 72 °C, and the final step lasted 30 min at 72 °C. For PCR Mix 2, the initial step was performed at 95 °C for 15 min, followed by 34 cycles each of 30 s at 94 °C, 90 s at 60 °C, and 60 s at 72 °C, and the final step lasted 30 min at 60 °C.

The amplified fragments were separated and sized by performing capillary electrophoresis with a CEQ 8000 Genetic Analysis System (Beckman Coulter Inc., Fullerton, CA, USA), applying a voltage of 6 kV at a temperature of 50 °C, with a capillary length of 30 cm, for 35 min [15].

#### 2.4.4. Statistical Analysis

Genetic analysis was performed using the poppr (v2.8.3) package of R statistical software v3.6.1 (Vienna, Austria) [16]. Dendrograms were created using Nei’s distance and UPGMA clustering.

In a previous work, the distance found between isolates of the same ADY allowed us to define the level at which two isolates can be considered as coming from the same strain [17]. In the fermentation of strawberry tree fruit, as no ADYs are used, the microsatellite profiles of two commercial wine ADYs were included in the genetic analysis, namely ADY CE and BDX, in order to serve as a reference for the distance between isolates from the same strain.

### 2.5. Distillation Description and Distillate Sampling

After the completion of the alcoholic fermentation, the fermented mass from each container in study was individually distilled in an industrial copper alembic, applying the cutting, that is, the separation of the different fractions of the distillation, namely the heads, hearts, and tails. Only the heart fractions (the spirits) were sampled to evaluate their volatile compositions. The fermented fruit from P1 was distilled in a traditional alembic system, with a wood heating system and a discontinuous steam distillation process. The first part, with approximately 5% (above 70% vol., with a strong, pungent, and unpleasant flavor) of the distillates, was collected as the head fraction. The heart fractions, obtained by single distillation, were collected when the ethanol concentration varied from 70 to 35% *v/v*; finally, the tail fractions were obtained when the alcoholic content decreased below 35% *v/v*, as previously described [18]. The fermented mass of the fruit from P2 was distilled in a modern copper alembic system, with a wood heating system and a steam distillation system coupled with a fractionation (rectification) column with 20 plates.

The distillates produced from each container were identified, as shown in Table 3. About 20 glass bottles of 0.75 L were filled with the distillate from each container. Two bottles of each distillate (two replicates) were submitted to further analyses.

### 2.6. Analytical Distillate Determinations

To evaluate the distillates, various analytical determinations were performed. Alcoholic strength (*v/v*)—expressed as total alcohol by volume (TAV)—was assessed by electronic densimetry (OIV, 2014). The pH was evaluated by potentiometry (OIV, 2014). Volatile quantification of the methanol, ethyl acetate, acetaldehyde, and fusel alcohols of the distillation fractions were performed with a gas chromatograph attached to a flame ionization detector (GC–FID), using Agilent 6890 GC equipment (Agilent Technologies, Wilmington, DE, USA), according to the method validated previously [19]. The quantification was done by analyzing the corresponding standard compounds with the same chromatographic conditions.

The identification of volatile compounds was carried out by gas chromatography connected to mass spectrometry (GC–MS) with equipment (Magnum, Finnigan Mat, San Jose, CA, USA) fitted out with an INNOWAX capillary column (30 m × 0.25 mm × 0.25 μm; J&W, Folsom, CA, USA), working under similar conditions as the GC–FID. The oven program was analogous to that used for GC–FID measurements. Mass spectra were obtained in the electron impact (EI) mode (ionization energy, 70 eV) and in full-scan mode (mass range *m/z* 20–340). The identification was done by comparing the mass with the NIST library and by analyzing the mass spectra of standards.

#### Standards and Chemicals

Ethanol and methanol were purchased from Merck (Darmstadt, Germany). GC–FID standards: ethyl acetate (CAS N° 141-78-6; purity ≥ 99.8%) and acetic acid (CAS 64-19-7; purity 99.8%) were purchased from Riedel-de-Haen (Seelze, Germany), methanol (CAS N° 67-56-1; purity ≥ 99.9%) was purchased from Merck (Darmstadt, Germany). 2-methylbutan-1-ol (CAS N° 137-32-6; purity ≥ 98%), 3-methylbutan-1-ol (CAS N° 123-51-3; purity ≥ 98.5%), butan-1-ol (CAS N° 71-36-3; purity ≥ 99.5%), 2-methylpropan-1-ol (CAS N° 78-83-1; purity ≥ 99.5%), propan-1-ol (CAS N° 71-23-8; purity ≥ 99.5%), 2-propen-1-ol (CAS N° 107-18-6; purity ≥ 98%), butan-2-ol (CAS N° 78-92-2; purity ≥ 99.5%), 4-methylpentan-2-ol (CAS N° 108-11-2; purity ≥ 98%), and acetaldehyde (CAS N° 75-07-0; purity ≥ 99.5%) were purchased from Fluka (Buchs, Switzerland).

### 2.7. Sensory Analysis of Distillate Samples

#### 2.7.1. Sample Preparation and Presentation

Given the high alcoholic content of the samples from Producer 2, they were diluted until they reached an alcoholic content of 42% *v/v* prior to the sensory evaluation. The samples of *arbutus* spirit were served (30 mL) at ambient temperature (21 °C) in wine glasses [20], labeled with three-digit codes, and evaluated in individual booths at the sensory room of INIAV, in Dois Portos, Portugal.

#### 2.7.2. Sorting Task

The samples were presented in a balanced order [21] to 10 tasters with experience in the sensory evaluation of wine, *arbutus*, mark, and honey spirits. Each taster received eight glasses with the eight samples of the arbutus spirits. In the first step, the tasters were asked to sort the samples into groups based the similarities between their odors, as determined by sniffing. They were permitted to make as many groups as they wished, which could range from 1 (if they thought that all of the samples were the same) to 8 (if they thought that all the samples were different). Once this sorting task was completed and they had made their groupings, the tasters were asked to proceed with a similar sorting task based on flavor (in-nose and in-mouth sensations).

For each taster, the results of the sorting task were organized into distance matrices, where the rows and columns correspond to the arbutus spirits. For each individual matrix, a value of 0 or 1 indicates that the samples were in the same or different groups, respectively, as described by other authors [22]. The individual matrices were summed across the tasters, resulting in a matrix of co-occurrences, which represents the global similarity between the samples.

Correspondence analysis (CA) was performed on the matrix of co-occurrences, as proposed by other authors [22,23] so as to obtain a spatial representation of the samples.

## 3. Results and Discussion

### 3.1. S. cerevisiae Characterization and Differentiation

The strawberry tree fruit fermentations were carried out by two producers (P1 and P2) using fruit harvested in October and December.

When collecting the fermented mass for microbiological analysis, the temperature and Brix degrees were measured for each deposit, as shown in Table 4.

The fermented mass of fruit collected in October from Producer 2 appeared to be slightly advanced as compared to that of Producer 1, considering the Brix values at T1 and T2. On the other hand, the decrease in the Brix value during the fermentation process of fruit harvested in December was greater in relation to that of the fruit harvested in October for the analyzed period (T1–T2).

Plate-count analyses (total count, yeast, and acetic acid bacteria) were performed in duplicate. The colonies formed were counted, and average values are recorded in Table 5, where the elapsed times since the collection of the fruit in the deposits are also recorded. Both the total count and the yeast count presented similar results, at the level of 10^7^ colony-forming units (cfu) per milliliter during the fermentation period analyzed (5–140 days), similar to what has been observed by other authors [8,24]. When the values at the beginning of the fermentation are compared with those from the middle of the fermentation (samples F and J, and H and L), a slight increase in the total and yeast counts can be noticed. In samples taken while the fermentation was already taking place (samples E and I, and G and K), the opposite can be observed, with a small decrease in the number of colony-forming units. In relation to the acetic bacteria, only samples F and H presented colonies at a level of 10^6^ and 10^4^ per milliliter, respectively, which correspond to the beginning of fermentation. Nevertheless, they were not detected in the following sampling period (T2), which indicates that, presumably, these bacteria tend to disappear during fermentation.

In order to characterize the yeast isolates, 20 yeast colonies grown in yeast growth media (10 yeast colonies per replicate sample) were randomly selected. Each isolate was further purified in YPD media. After cell lysis, PCR with specific primers for *S. cerevisiae* was performed, followed by gel electrophoresis. The results obtained allowed the presumptive differentiation of the *S. cerevisiae* isolates from the non-*Saccharomyces cerevisiae* isolates, depending on the detection of the amplicon as a positive result for *S. cerevisiae* (Table 6).

From a total of 160 isolates, 94 were characterized as *S. cerevisiae* species. In the case of isolates from samples F and H, no *S. cerevisiae* were detected, most probably because the isolates were obtained from samples collected only 2 and 5 days after harvest. Thus, the initial phase of fermentation seems to be characterized by a predominance of non-*Saccharomyces* yeasts, similar to what occurs in wine fermentation [25,26], corroborating the low numbers of *S. cerevisiae* species found on the surfaces of *Arbutus unedo* fruit [8].

The yeast isolates that tested positive for *S. cerevisiae* species were further differentiated using microsatellite primers (SSR). The microsatellite profiles of C3, C5, C8, C11, and SCAAT3, C4, C6, YKL172, ScAAT1, ScAAT5, and YPL009c SSRs were determined using two multiplex reactions followed by capillary electrophoresis in an automatic Beckman Coulter DNA Sequencer.

**Figure 1:**
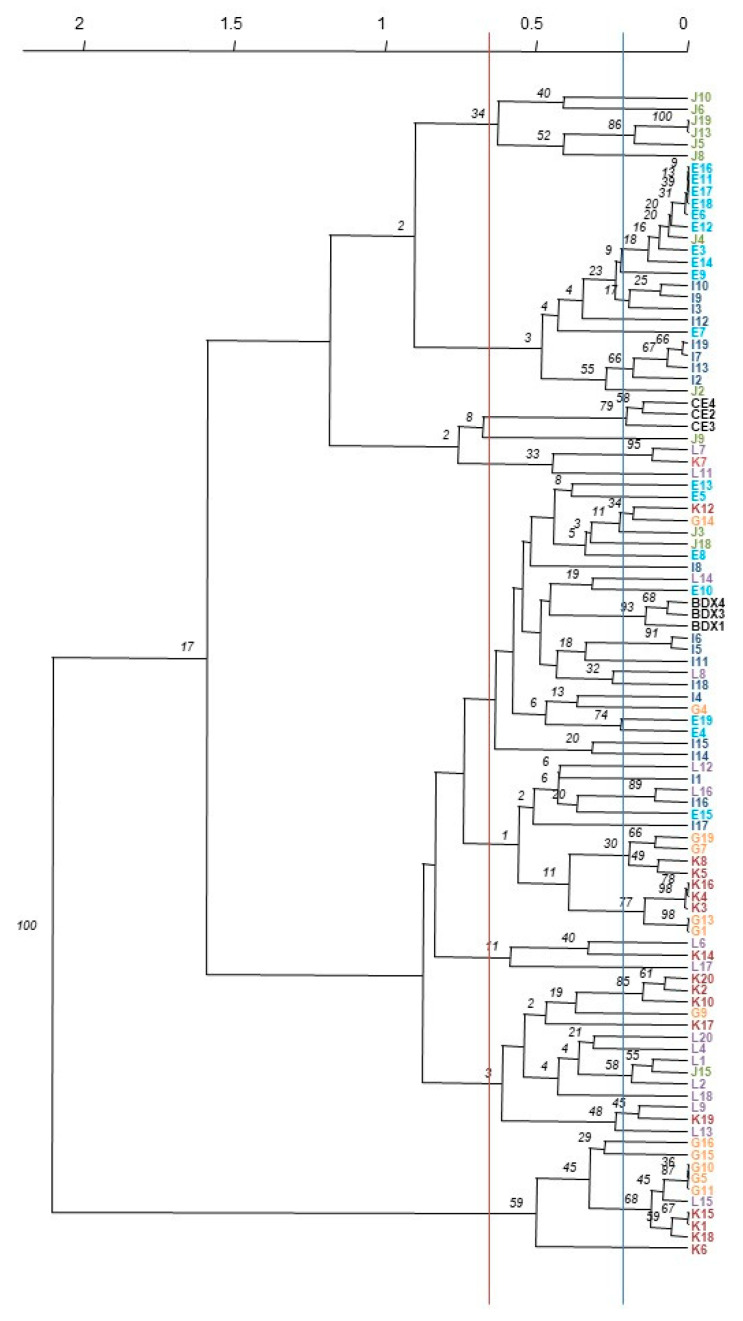
Dendrogram presenting the genetic distance between the *S. cerevisiae* isolates from *Arbutus unedo* fermentations (codes in Table 6) and ADY isolates (codes CE and BDX) based on microsatellite profiles. The scale at the top represents genetic distance. The blue line and the red line across the dendrogram represent the strain and the closest genetic levels, respectively.

The dendrogram established using the poppr (v2.8.3) package of the R statistical software is represented in Figure 1.

If we consider the distance between the commercial, active dry yeast isolates, each of the three corresponding to one strain, around 56 different *S. cerevisiae* strains were detected among the 94 isolates from the *Arbutus unedo* fermentation samples (the blue line across the dendrogram.

Another line was drawn along the dendrogram (red line), and eight major clusters were obtained. Cluster 1 groups 6 isolates from P1 detected in fermentation samples from fruit collected in December (4 strains). Cluster 2 also groups 20 isolates from P1, but from fermented samples of fruit harvested in October (codes E and I). Three strains could be differentiated at each sampling. In this cluster, there is the exception of isolates J2 and J4, as they are from the deposit of fruit collected in December. The ADY CE groups separately. Cluster 3 comprises isolates from P2, detected in T2 samples, the isolate from fermented October fruit (K7) being similar to a strain (L7) from December’s fermented fruit. Cluster 4, comprising 21 isolates (from E3 to I14), corresponds to a high number of strains (19), grouping both producers, fruit harvests, and sampling times. Cluster 5 groups 18 isolates from both P1 and P2, most probably corresponding to only 7 strains. A total of 2 sub-clusters can be observed, one of them with isolates only from P2, that correspond to 2 strains from October’s fruit harvest (G and K). Cluster 6 groups 3 strains from P2 at T2, from both the October and December fruit harvests. Cluster 7 groups isolates from P2, the majority coming from December’s fermented fruit, sampled at T2 (7 isolates corresponding to 6 strains), 6 isolates from October’s fermented fruit, sampled at T2 (3 strains), and 1 strain from the T1 sampling time (G9). There is 1 isolate from P1 which also grouped in this cluster (J15). Cluster 8 is the most distinct cluster, with 10 isolates coming only from P2 and corresponding to 4 strains, 1 of them being common to both harvests and both sampling times (isolates G10, G5, G11, L15, K15, K1, and K18).

In general, and despite the great diversity encountered in the 94 isolates differentiated by SSR markers, there is a good separation between isolates from P1 and P2. Isolates from P1 group in Clusters 1 and 2. Clusters 3, 6, and 8 group isolates from P2. Cluster 7 also groups isolates from P2 with only 1 isolate from P1. Only 2 groups encompass isolates from both producers.

The separation of the harvesting times was not that clear. Only Cluster 1 grouped isolates from the December harvest period for P1. Nevertheless, the other isolates from this sampling (J) are scattered all over the dendrogram, grouping in Clusters 2, 4, and 7. Cluster 2 encompasses mostly isolates from the October harvest in P1. In P2, all clusters contain isolates from both harvest times.

### 3.2. Distillates Characterization and Differentiation

**Figure 2:**
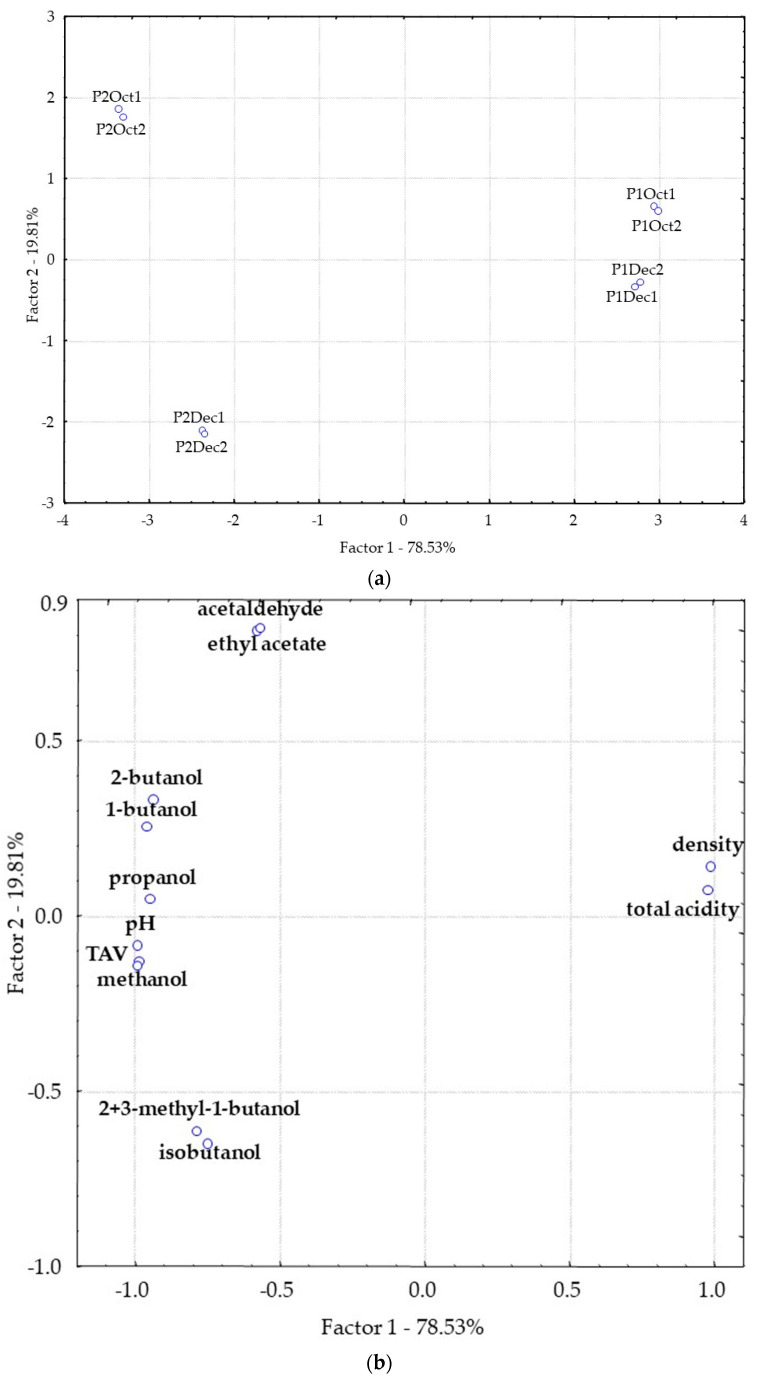
Plot of the distillate samples (**a**) and the variables (**b**) in the plane formed by the two first components from the PCA. Replicates are identified by numbers 1 or 2 after the sample code.

The results of the distillates analytical determinations were submitted to principal component analysis (PCA). The plot of the samples on the plane of the first two components, which explain 78.53 and 19.51% of the variance, is shown in Figure 2.

The first component allowed the greatest separation of the distillates from the two producers, with samples from Producer 1 located on the right side of component 1 and samples of the P2 on the opposite side of this component. The projection of the variables in the same plane showed that the variables with the greatest contribution to this separation are acidity, pH, density, TAV, methanol, 2-butanol, 1-propanol and 1-butanol. The samples from P2 are more related to higher levels of TAV, methanol, 2-butanol, 1-propanol, 1-butanol, and pH and with lower levels of density and acidity.

There seems to be a slight separation of the distillation samples along component 2 which seems to be related to the harvest time. Actually, the samples produced with the fruit harvested in October are located on the positive side of this component, which seems to be related to higher amounts of acetaldehyde and ethyl acetate, while the samples produced with fruit harvested in December are located on the negative side of this component more related to higher amounts of isobutanol.

**Figure 3:**
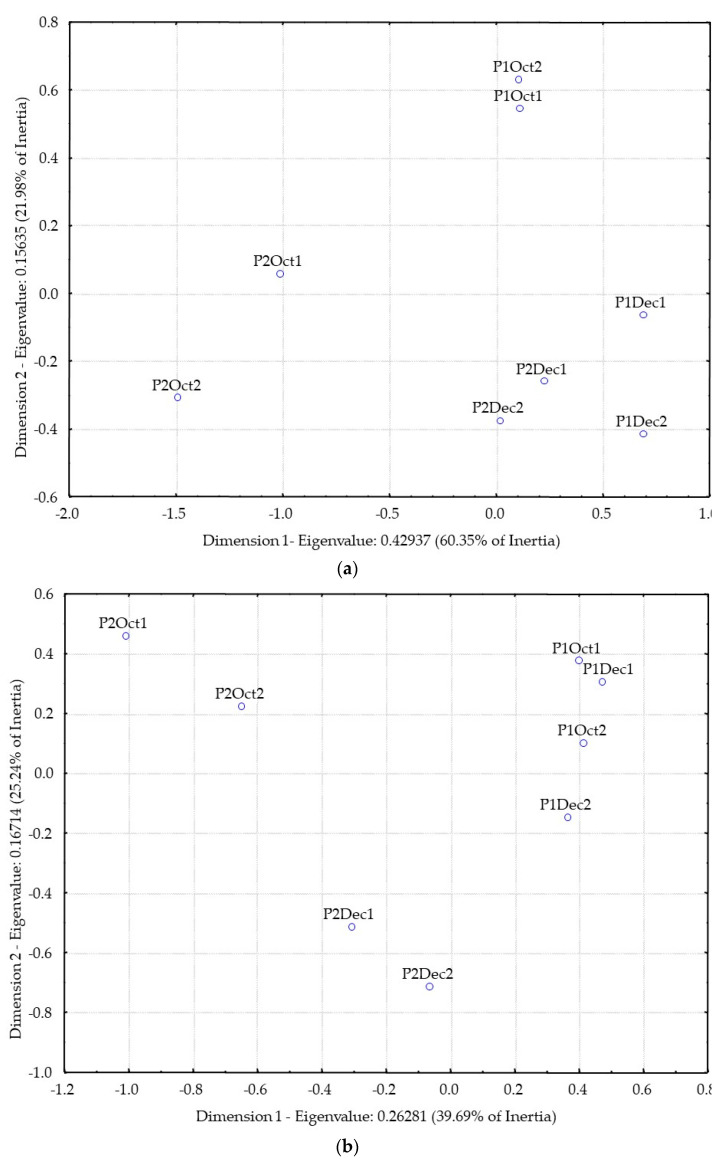
Distillates configuration from Correspondence Analysis applied to the odor co-occurrence matrix (**a**) and to flavor co-occurrence matrix (**b**). Replicates are identified by numbers 1 or 2 after the sample code.

Figure 3a shows the results of the correspondence analysis of the distillates odor similarity matrix. Figure 3b shows of the correspondence analysis of the distillates flavor similarity matrix.

From the analysis of Figure 3a, based on the odor similarities of the distillates, it is not clear the separation between producers (P1 and P2). However, there is a relatively high similarity of odor among distillates from December fruit harvest for both producers (P1 and P2).

Considering flavor analysis (Figure 3b), P1 distillates are closer together, but well separated from P2 distillates. Moreover, P2 distillates are separated from each other according to the different harvest time (October and December).

Therefore, the analysis of the physical-chemical and sensorial results highlighted a clear separation between the distillates of the two producers, in agreement with the microbiological results. Further research is needed to study the relationship between territory and products, as is being studied with other alcoholic beverages [27].

## 4. Conclusions

Microbiological analyses showed that a high level of yeasts was present in the fermenting fruit masses throughout the fermentation process, with most isolates characterized as *S. cerevisiae*. Although a great diversity was detected among the *S. cerevisiae* isolates, the analysis of the genetic distance allowed the clustering of the isolates, with a good separation between producers. The differentiation of the harvest period was not so clear. Only isolates from P1 were roughly clustered separately.

Similarly, the results of the analytical determinations and sensorial analysis showed a greater discrimination between producers and a slight influence of the harvest season, which may lead us to think about a possible connection between the chemical and sensorial characteristics of the distillates and the specific indigenous microbiota of each fermented product.

The characterization of the indigenous yeasts associated with the fermentation process of *Arbutus unedo* L. fruit can be an important contribution to the preservation of the specific characteristics of their spirits. This knowledge can be a differentiating factor for local products and can contribute to increasing the intrinsic value of the Portuguese spirit “Aguardente de medronho”.

In future works, we foresee the selection of autochthonous yeast isolates and their use in controlled alcoholic fermentations.

## Figures and Tables

**Table 1:** Biophysical characterization of the experimental areas.

Location	Farming Type	Year of Installation	Average Annual Temperature (°C)	Average Annual Rainfall (mm)	Soil Classification (WRB)
Estreito (P1)	Orchard from seedlings	2005	7.5–10.0	1200–1400	Cambisols
Signo Samo (P2)	Orchard from clonal plants	2014	12.5–15.0	1200–1400	Dystric Leptosols

**Table 2:** Sampling periods and codes used in yeast isolates.

Date of Harvest	Sampling Period	Producer 1 (P1)	Producer 2 (P2)
October 2019	December 2019 (T1)	E (1–20)	G (1–20)
	February 2020 (T2)	I (1–20)	K (1–20)
December 2019	December 2019 (T1)	F (1–20)	H (1–20)
	February 2020 (T2)	J (1–20)	L (1–20)

**Table 3:** Harvest periods and distillate codes for each producer.

Date of Harvest	Producer 1 (P1)	Producer 2 (P2)
October 2019	P1 October (E,I) *	P2 October (G,K) *
December 2019	P1 December (F,J) *	P2 December (H,L) *

* codes corresponding to yeast isolates.

**Table 4:** Characterization of the fermented mass at the sampling periods (Brix degree and temperature).

Producer	Date of Harvest	Sampling Time	Sample Code	°Bx	Temperature (°C)
P1	31 October 2019	T1	E	19.8	14.2
		T2	I	16.2	14.0
P1	6 December 2019	T1	F	18.3	14.1
		T2	J	8.3	14.0
P2	23 October 2019	T1	G	17.0	13.0
		T2	K	14.1	13.0
P2	3 December 2019	T1	H	18.8	13.0
		T2	L	9.5	13.0

**Table 5:** Plate-count results (total microorganisms, yeasts, and acetic bacteria) in cfu/mL over fermentation (average of duplicate samples).

Producer	Sample Code	Sampling Period	Fermentation Days	Total Count (cfu/mL)	Yeast (cfu/mL)	Acetic Bacteria (cfu/mL)
	E	T1	38	2.4 × 10^7^	2.7 × 10^7^	<1 *
	I	T2	96	1.4 × 10^7^	1.5 × 10^7^	<1 *
P1	F	T1	2	5.5 × 10^6^	3.4 × 10^6^	1.7 × 10^6^
	J	T2	60	1.9 × 10^7^	1.8 × 10^7^	<1 *
	G	T1	46	1.8 × 10^7^	1.6 × 10^7^	<1 *
	K	T2	140	1.8 × 10^7^	1.5 × 10^7^	<4 *
P2	H	T1	5	1.2 × 10^7^	1.3 × 10^7^	7.4 × 10^4^
	L	T2	63	2.5 × 10^7^	2.5 × 10^7^	<4 *

* in 10^−2^ mL.

**Table 6:** *S. cerevisiae* and non-*Saccharomyces* isolates in each sample.

Code Samples	*S. cerevisiae* (Isolate Number)	Non-*Saccharomyces cerevisiae* (Isolate Number)
E	1, 3, 4, 5, 6, 7, 8, 9, 10, 11, 12, 13, 14, 15, 16, 17, 18, 19	2, 20
F		1–20
G	1, 4, 5, 7, 9, 10, 11, 13, 14, 15, 16, 19	2, 3, 6, 8, 12, 17, 18, 20
H		1–20
I	1–19	20
J	2, 3, 4, 5, 6, 8, 9, 10, 13, 15, 18, 19	1, 7, 11, 12, 14, 16, 17, 20
K	1, 2, 3, 4, 5, 6, 7, 8, 10, 12, 14, 15, 16, 17, 18, 19, 20	6, 11, 13
L	1, 2, 4, 6, 7, 8, 9, 11, 12, 13, 14, 15, 16, 17, 18, 20	3, 5, 10, 19

## Data Availability

Data is contained within the article.
